# Transient enhancement of proliferation of neural progenitors and impairment of their long-term survival in p25 transgenic mice

**DOI:** 10.18632/oncotarget.9834

**Published:** 2016-06-06

**Authors:** Donghua Zou, Yijing Zhou, Long Liu, Fengping Dong, Tianzhi Shu, Ying Zhou, Li-Huei Tsai, Yingwei Mao

**Affiliations:** ^1^ Department of Neurology, The Fifth Affiliated Hospital of Guangxi Medical University & The First People's Hospital of Nanning, Nanning, Guangxi, China; ^2^ Department of Biology, Pennsylvania State University, University Park, PA, USA; ^3^ Department of Chemistry and Biology, College of Science, National University of Defense Technology, Changsha, Hunan, China; ^4^ Picower Institute for Learning and Memory-MIT, Cambridge, MA, USA

**Keywords:** p25, CDK5, neurogenesis, transgenic mice, neural progenitor, Gerotarget

## Abstract

Cyclin-dependent kinase 5 (CDK5) regulates important neuronal functions via p35. p35 undergoes cleavage in response to neuronal activity and neurotoxic conditions to release its subunit p25. Although p25 has been implicated in various neurodegenerative diseases, the mechanisms by which p25 mediates neurodegenerative impairment have not been fully elucidated. We aimed to determine the role of p25-mediated neurodegeneration on neurogenesis in an inducible transgenic mouse line overexpressing p25 (p25 TG) in the forebrain. Adult neuronal progenitor cells (NPCs) were labeled with BrdU *in vivo*, which were significantly increased in numbers in the subventricular zone, the hippocampus, and the cortex of p25 TG mice. Consistently, more mitotic cells were observed in p25 TG mice than in controls, even in the cortex and the CA1, which are not neurogenic regions. BrdU-positive cells were negative for GFAP or γ-H2AX, suggesting that they are not astrocytes or dying cells. Neurospheres derived from the dentate gyrus and the cortex were significantly increased in p25 TG mice and can be differentiated into astrocytes and neurons. However, p25 TG decreased the long-term survival of proliferating NPCs and severely impaired adult neurogenesis. A Transwell co-culture system was used to assess the influence of p25-expressing primary neurons on adult NPCs. Co-culture with p25-expressing neurons downregulated Ki67 expression and upregulated cleaved caspase-3, indicating that the paracrine signaling in cell-cell communication is essential for NPC survival and proliferation. Moreover, increased CDK5 activity impairs Wnt activation. This study demonstrates that hyperactivation of p25 may temporarily enhance NPC proliferation, but impair their long-term survival.

## INTRODUCTION

Cyclin-dependent kinases (CDKs) are a group of protein kinases with crucial roles in regulating the cell cycle [1]. CDK5, a member of the CDK family, is predominantly expressed in neurons of the mammalian brain and mediates diverse cellular events during neuronal development and function, including synaptogenesis [2], various neurodegenerative diseases [3], neuritic outgrowth [4, 5], growth cone maintenance [6], mitochondrial function [2], and microtubule architecture [7]. CDK5 activity also is required for the migration and differentiation of normal neurons [8].

The activity of CDK5 depends on the binding of its activator, p35 [9, 10]. p35 is highly expressed in post-mitotic neurons, but absent from proliferating neuronal progenitors [11]. Downregulation of p35 has been shown in various disease models, such as the valproic acid animal model of autism [12], and is crucial for cortical development [10, 13]. Calpain-mediated proteolytic cleavage of p35 yields the p25 fragment, which has a longer half-life and more diffuse subcellular distribution than p35. p35 is bound to the membrane, whereas p25 is localized to the cell soma and nucleus [14, 15]. Thus, p25/Cdk5 has properties that are distinct from p35/Cdk5. The Cdk5/p25 association is longer and uncontrolled, causing aberrant hyperphosphorylation of various Cdk5 substrates, such as amyloid precursor protein, tau, and neurofilaments, leading to neurodegenerative pathology, including Alzheimer's disease (AD) [16–19]. Accumulation of p25 may contribute to neurotoxicity and neuronal death in AD pathology by inducing abnormal CDK5 activity and subsequently triggering inappropriate cell-cycle re-entry in mature neurons [8, 20, 21]. A number of reports have described a correlation between neuronal activity and Aβ production [22, 23], and p25 overexpression increases Aβ levels *in vivo via* a mechanism involving BACE1 [24, 25]. p25 accumulation causes neurodegeneration in pathological conditions, but the precise role of p25-induced neurotoxity in neuronal development and differentiation has not yet been fully clarified [26].

In the present study, we established an inducible transgenic mouse line that overexpresses p25 (p25 TG) in the postnatal forebrain. In the adult brain, neurogenesis is assumed to occur in two main regions, the subgranular zone (SGZ) in the hippocampal dentate gyrus (DG) and the subventricular zone (SVZ) of the lateral ventricle [27]. Hence, in this study, neuronal progenitor cell (NPC) proliferation, differentiation, and death were compared in these regions in wild-type (WT) and p25 TG mice. *In vitro* growth and differentiation of neurospheres derived from these regions were also examined. In addition, the potential pro-apoptotic effect of p25 was determined in primary neurons expressing p25 in a neural stem cell co-culture system. Our findings provide basic evidence for understanding the functional role of p25 in regulating neurogenesis under pathophysiological conditions. Moreover, strategies targeting the modulation of p25 in the mammalian brain may facilitate neuro-regeneration in neuronal pathology.

## RESULTS

### Proliferation of NPCs in p25 TG mice

**Figure 1:**
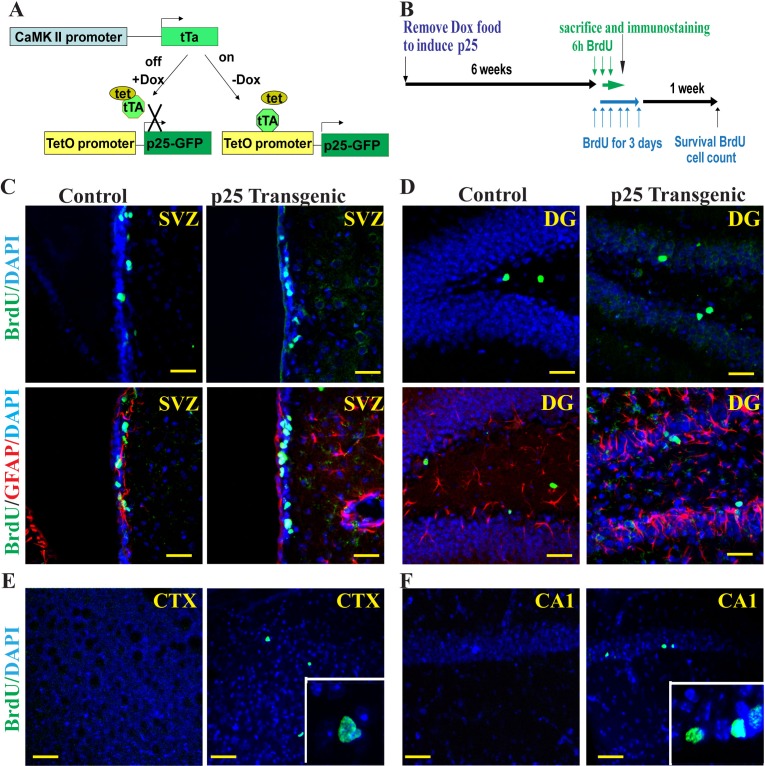
Proliferation of neural progenitors BrdU and GFAP double immunofluorescent staining was used to label proliferating neurons in p25 transgenic mice (p25 TG). The bitransgenic CK-p25 Tg mouse expresses p25 under the control of the CaMKII promoter, which can be switched on or off with a doxycycline diet (**A**). After 6 weeks of induction, BrdU was pulsed-injected to label the proliferating cells (**B**). Four brain regions were examined: the SVZ (**C**), the DG (**D**), the cortex (**E**) and the CA1 regions (**F**). The inserts in panels E and F show the overlap of BrdU staining with DAPI. More proliferating neural progenitors were observed in p25 TG than in control mice. BrdU (green) and GFAP (red) double-labeling showed that BrdU-positive neurons were not GFAP-positive (**C**, **D**). SVZ, the subventricular zone; CTX, the cortex; DG, the hippocampal dentate gyrus. Blue: DAPI staining. Scale bar = 20 μm.

To determine the p25-mediated cell toxicity on neurogenesis, we established a bitransgenic mouse line in which inducible green fluorescent protein (GFP)-tagged human p25 expression is controlled by the CamKII promoter (CK)-regulated tet-off system. In bitransgenic CK-p25 mice, p25 expression is repressed in the presence of doxycycline [28]. To induce p25 production, mice were taken off the doxycycline diet for 6 weeks (Figure 1A). These mice develop neurodegeneration and tau-associated pathology upon p25 induction. To further elucidate the effects of p25-induced neurodegeneration, p25 TG mice were injected with BrdU at 50 μg/g body weight, every 2 hours for three times, and animals were sacrificed 2 hours after the last injection (Figure 1B). We counted the BrdU-positive neurons in the SVZ (Figure 1C), DG (Figure 1D), cortex (Figure 1E), and CA1 region (Figure 1F) of transgenic and control WT mice. Double immune-fluorescent staining revealed that almost all of BrdU-positive cells in the SVZ and DG of WT mice or in the SVZ, DG, cortex, and CA1 of p25 TG mice were not positive for glial fibrillary acidic protein (GFAP) (Figure 1C-1D, supplementary Figure S1A-S1B). This result is consistent with the theory that short-term BrdU labels transient amplifying progenitors instead of slowly dividing neural stem cells (NSCs). Neither were BrdU-positive cells in the hippocampus and cortex positive for GFAP in the p25 mice, in which GFAP was drastically upregulated. This suggested that, at the time examined, these proliferating cells were unlikely generated by reactive gliosis.

**Figure 2:**
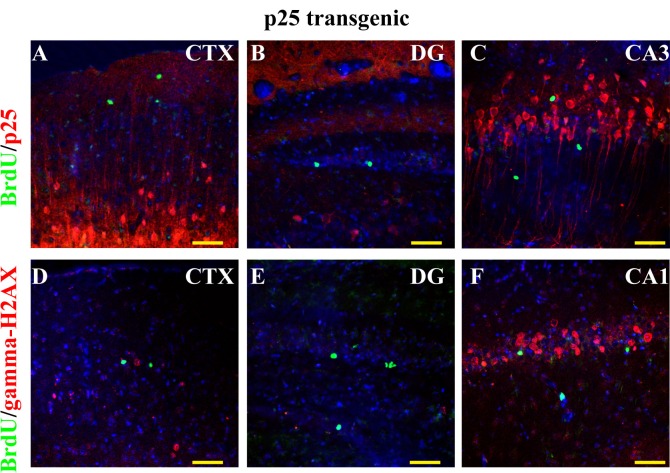
BrdU labeling and cell-cycle re-entry No BrdU-positive cells were p25-EGFP- positive in the CTX (**A**), the DG (**B**), or the CA regions (**C**). No co-localization existed between BrdU and γ-H2AX, where γ-H2AX was used as a marker for DNA damage caused by cell-cycle re-entry (**D** to **F**). Blue: DAPI staining. Scale bar = 50 μm.

GFP-p25 was expressed in the forebrain excitatory neurons. We found that BrdU-positive cells in p25 TG mice did not express p25 (Figure 2A-2C), indicating that they are not derived from neurons that re-entered the cell cycle in response to the transgene p25-induced DNA damage. Additionally, using γ-H2AX to label DNA damage, we found that high levels of DNA damage occurred in the cortex and CA1, but much less in DG regions (Figure 2D-2F). The level of γ-H2AX correlated with the level of p25 expression, supporting the idea that p25 has a key role in causing DNA damage. BrdU-positive cells were not γ-H2AX-positive (Figure 2D-2F), indicating that BrdU incorporation in those cells was not caused by DNA damage.

**Figure 3:**
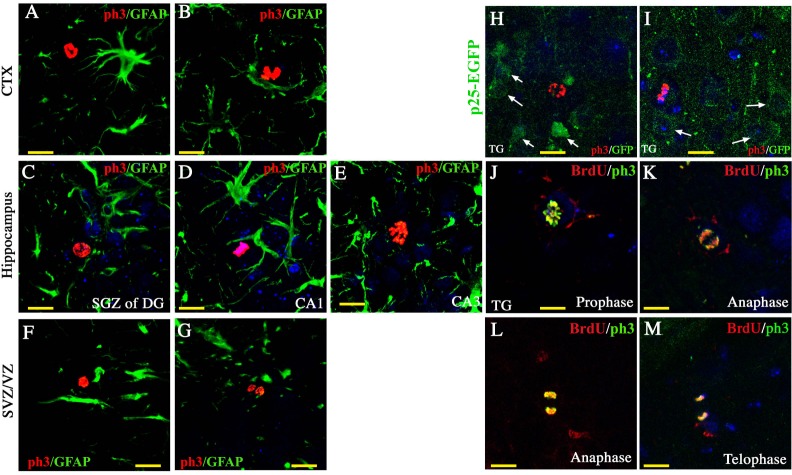
Cell mitosis of neural progenitors Using ph3 as a specific marker for mitotic neural progenitors, we found proliferating cells in the cortex (**A**, **B**), the hippocampus (**C** to **E**), and the SVZ (**F** and **G**). These proliferating cells were not GFAP-positive or p25-EGFP- positive (**H** and **I**). Co-localization of BrdU and ph3 was observed at all four phases of mitosis (**J** to **M**). Blue: DAPI staining. Scale bar = 10 μm.

Phospho-histone H3 (ph3) was used to label cells at M phase (Figure 3). Intriguingly, ph3-positive proliferating cells were detected in the neurogenic regions, such as the DG of the hippocampus (Figure 3C) and the SVZ (Figure 3F-3G), and also in the non-neurogenic regions, including the cortex (Figure 3A-3B, 3H-3I), the CA1 (Figure 3D), and the CA3 of the hippocampus (Figure 3E). Consistent with the result from BrdU labeling, ph3-positive cells in p25 TG mice were positive for neither GFAP (Figure 3A-3G) nor p25-EGFP (Figure 3H-3I), suggesting that they are unlikely derived from either the dividing reactive glial cells or transgene-expressing neurons. Moreover, those cells were co-labeled by both BrdU and ph3 at all four stages of the mitotic cell cycle, confirming that BrdU labeled the dividing neural progenitors *in vivo* (Figure 3J-3M).

### Quantification of neurogenesis

**Figure 4:**
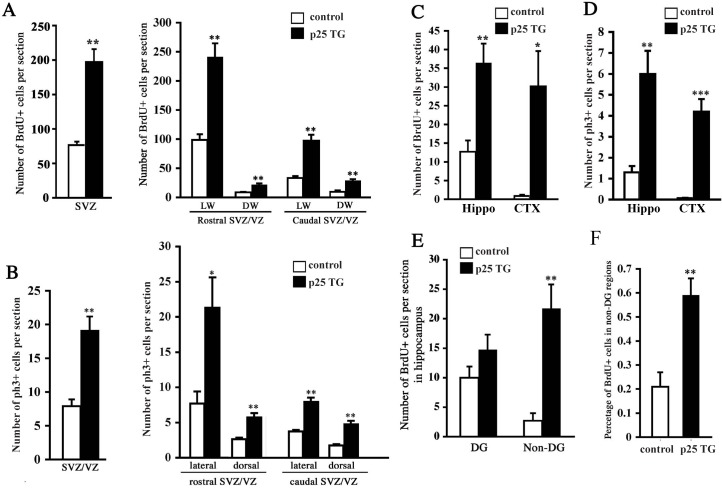
Quantification of neurogenesis in p25 transgenic mice The numbers of BrdU-positive cells in the SVZ (**A**), the hippocampus and the cortex (**C** and **E**) were significantly greater in p25 transgenic mice than in control mice. The number of ph3-positive cells was also increased in the hippocampus and cortex (**D**). More cells in the SVZ were at M phase in p25 transgene mice (**B**). Percentage of BrdU-positive cells in the non-DG regions (CA1 and CA3) *versus* the whole DG was significantly higher in p25 transgenic mice than in control mice (**F**). *, *p* < 0.05; **, *p* < 0.01 compared to WT (*n* = 3-7).

To further determine the actual change of cell proliferation in response to p25 insult, the number of proliferating (BrdU-positive) cells was quantified. Over 2.5-fold more BrdU-positive cells were observed in the SVZ of p25 TG mice (Figure 4A). As neurogenesis in different portions of the SVZ lining the lateral ventricle is not uniform, we further divided the SVZ into sub-regions, including the lateral walls (LW) and dorsal walls (DW), both of which can be further divided into rostral and caudal portions, using the boundary of the emerging point of hippocampal proper as a landmark. Cell proliferation was elevated in all sub-regions (Figure 4A). We obtained consistent results when using ph3 as an alternative marker for cell mitosis (Figure 4B) in the SVZ/VZ. Similarly, we observed more than a 2.5-fold increase of BrdU-positive cells in the hippocampus of p25 TG mice (Figure 4C). Intriguingly, a greater than 30-fold change was detected in the cortex, a non-neurogenic region (Figure 4C). The proliferative changes in the hippocampus and cortex were confirmed by the mitotic marker, ph3 (Figure 4D). Notably, proliferating cells in the DG, a well-known neurogenic region, did not show significant difference between control and p25 TG mice. However, BrdU-positive cells in the non-DG regions (the CA1 and CA3) of the hippocampus were significantly increased in p25 TG mice (Figure 4E). Even taking into account of the total BrdU-positive cells, the non-DG regions of p25 TG mice still exhibited higher percentage of proliferative cells (Figure 4F).

### Survival and fate determination of proliferating neural cells

**Figure 5:**
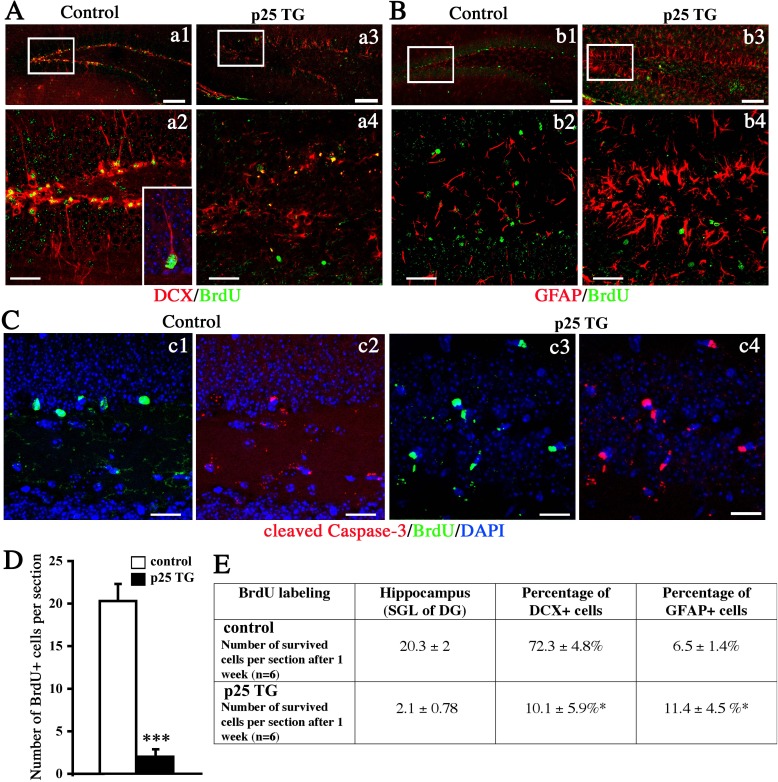
Differentiation and survival of proliferating neural cells After 1 week of survival, BrdU was co-labeled with DCX (**A**), GFAP (**B**), or activated caspase-3 (**C**). Figure **a1, a4, b2** and **b4** show the zoom-in boxes in **a1, a3, b1**, and **b3** respectively. Scale bar = 50 μm (**a1, a3, b1, b3**). Scale bar = 30 μm (**a2, a4, b2, b4** and **C**). **D.** Quantification of surviving cells in the hippocampus. ***, *p* < 0.005, *n* = 6. **E.** Development of the neural fate of BrdU-positive cells.

Next, we examined the viability of these proliferating neural cells 1 week after the short-term pulse-labeling with BrdU (6-h labeling, Figure 1B). A significantly higher percentage of BrdU-positive cells survived in WT mice than in p25 TG mice (data not shown). This observation is consistent with that cell death is ongoing in the newly born neural cells. Because the number of BrdU-labeled cells was rather low in the short-term labeling experimental paradigm above, we used a prolonged paradigm by injecting BrdU at a concentration of 50 μg/g body weight twice per day for 3 days (3-day labeling, Figure 1B). The fate of BrdU-positive cells was further examined by labeling with doublecortin (DCX) and GFAP after 1 week. About 72.3% proliferating NPCs differentiated into DCX-positive neurons normally in WT mice (Figure 5a1-a2, 5E). However, in p25 TG mice, only 10.1% BrdU-positive cells were differentiated into newborn neurons, and they showed unhealthy morphologies with the condensed or collapsed dendritic shape (Figure 5a3-5a4, 5E, supplemental S2). On the other hand, p25 TG mice had about two folds more astrocytes derived from BrdU-positive NPCs than WT mice (Figure 5B, 5E), suggesting that p25 expression in mature neurons changes the cell-fate specification of NPCs nearby.

As neurogenesis was remarkably decreased by p25, we next measured the level of apoptosis by activated caspase-3. Few BrdU-positive cells were co-labeled for activated caspase-3 in WT mice (Figure 5c1-5c2). In contrast, most BrdU-positive cells in p25 TG mice were positive for activated caspase-3(Figure 5c3-5c4), suggesting that p25 expression increases apoptosis of newborn neurons. We further compared the number of surviving BrdU-positive cells in the hippocampal dentate gyrus region. Consistently, about 10-fold more BrdU-positive cells were alive in the hippocampus of the WT mice (20.3±2) than in the TG mice (Figure 5D-5E). These data confirmed the results from the BrdU short-term experimental paradigm, showing that there was a decreased survival rate of dividing neural cells in the p25 TG mice despite an increase of transient proliferation.

### Neurosphere formation *in vitro*

**Figure 6:**
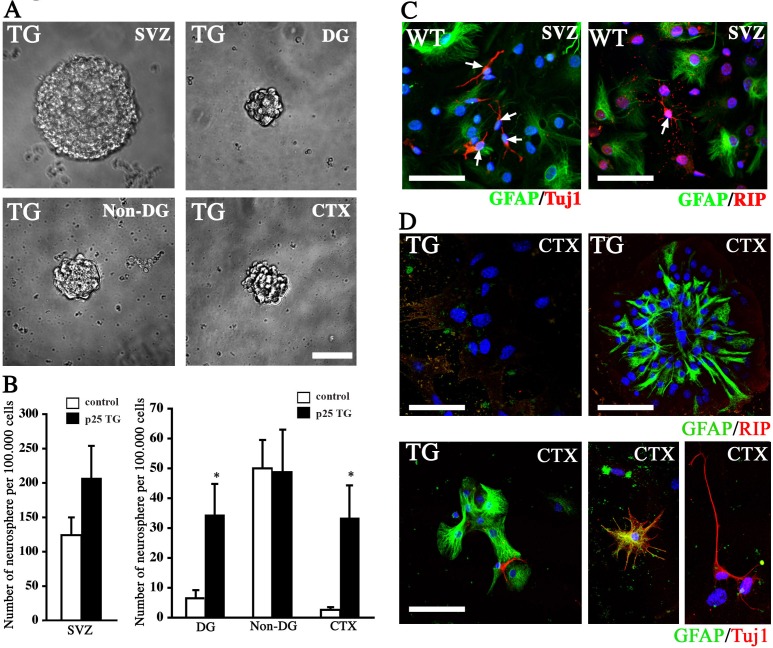
Neurosphere assay **A.** Representative neurospheres formed from the neural cells dissociated from different brain regions of p25 TG mice. Scale bar = 50 μm. **B.** Quantification of the number of neurospheres from different brain regions of WT and p25 TG mice. *, *p* < 0.05; *n* = 3. **C.** Different neural cell lineages into which SVZ neurospheres were differentiated. Scale bar = 30 μm. **D.** Neurospheres formed from the neural cells dissociated from the cortex of p25 TG mice brain also differentiated into three neural lineages. Blue: DAPI staining. Scale bar = 30 μm.

To further confirm that BrdU-labeled cells are truly multipotent NPCs, we performed an *in vitro* neurosphere assay to substantiate the *in vivo* findings. Specific regions were carefully dissected out from the brains of WT and p25 mice. These included the neocortex, the SVZ/VZ lining the lateral ventricles, and the hippocampal DG and non-DG regions. Cells from these regions were dissociated and grown free-floating in the conventional neurosphere culture medium. A week later, the number of neurospheres were counted, standardized and compared between different regions. Significantly more neurospheres were found in the DG and cortex of p25 TG mice than in WT mice (Figure 6A and 6B). No significant difference in the number of neurospheres was observed, however, in the SVZ and the non-DG regions of the hippocampus (the CA1 and the CA3). Collectively, the neurosphere assay supports the hyperproliferation of neural cells in p25 TG mice, especially in non-neurogenic regions, such as the cortex.

To further confirm that neurospheres generated from the cortex of the p25 TG mice contained multipotent NPCs, we dissociated the neurospheres and cultured them under differentiation-inducing conditions. Among the progeny of the cells derived from the SVZ/VZ neurospheres, 85 of 727 (11.69%) cells and 90 of 682 (13.20%) cells showed differentiation in WT and p25 TG mice, respectively. Most of these differentiated cells were positive for GFAP; a few cells were positive for TuJ1 or RIP1 (Figure 6C). In the cortex of p25 TG mice, 35 of 510 (6.86%) cells showed differentiation. Like those in the SVZ/VZ region, most cells differentiated into GFAP-positive cells; a few cells were TuJ1-positive (Figure 6D), indicating that they can differentiate into neuronal and glial lineages. However, no RIP-positive cells were found, suggesting that the oligodendrocyte lineage is limited.

### *In vitro* proliferation and survival of neural progenitors

**Figure 7:**
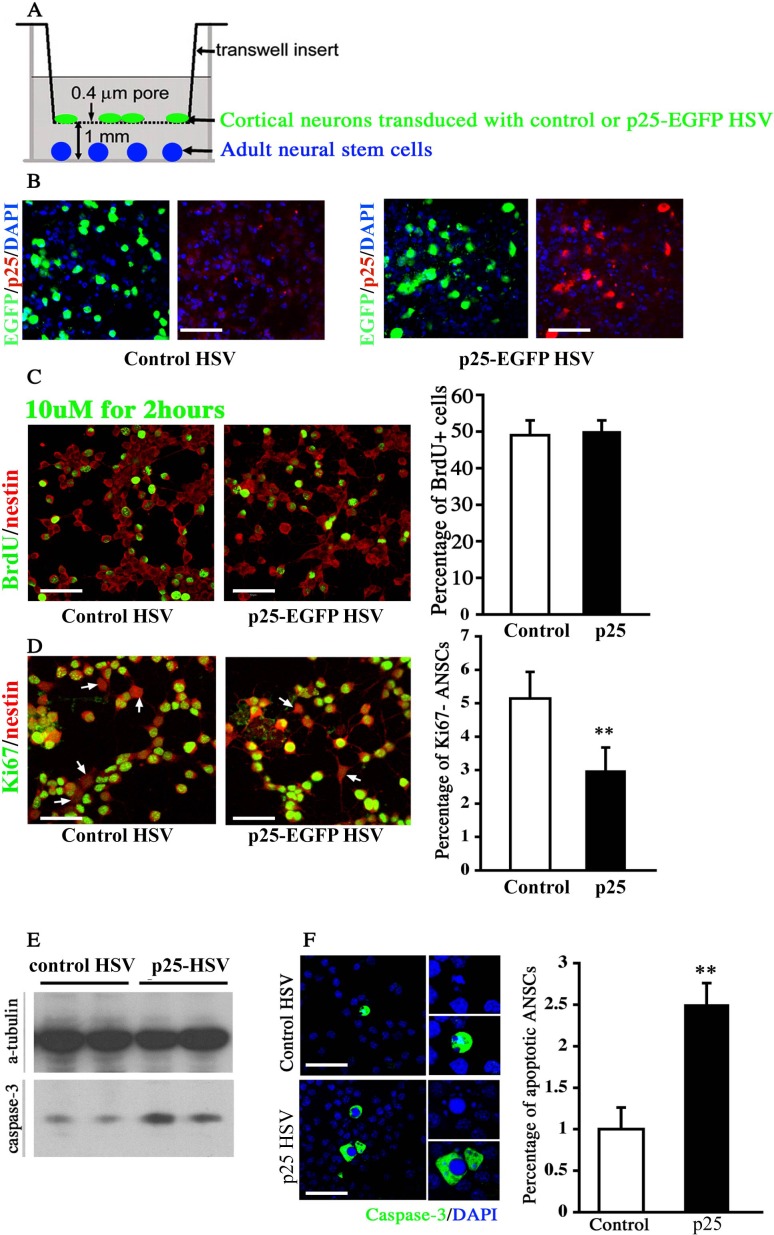
Co-culture assay of neural stem cells with p25-overexpressing neurons **A.** Schematic illustration of the co-culture assay. Cortical or hippocampal neurons were transfected with the herpes simplex virus (HSV) vector expressing control EGFP or p25-EGFP gene fragments. **B.** Co-labeling of cultured neural cells expressing the GFP and p25 genes. Scale bar = 30 μm. **C.** The expression level of BrdU was not changed after 1 day of co-culture with p25-overexpressing neurons. Scale bar = 30 μm. *n* > 3. **D.** The percentage of Ki67-negative cells decreased after 1 day of co-culture with p25-overexpressing neurons. Scale bar = 30 μm. **, *p* < 0.01; *n* > 3. **E.** Caspase-3 levels increased after 3 days of co-culture with the p25-overexpressing viral vector. **F.** The number of apoptotic cells was increased after 3 days of co-culture with the p25-overexpressing viral vector. Scale bar = 30 μm. **, *p* < 0.01; *n* > 3.

We performed an *in vitro* Transwell co-culture assay to directly test the effect of p25-induced neuronal death on the proliferation and the survival of NPCs (Figure 7A-7B). In this assay, dissociated cortical neurons were cultured on an inserted membrane with 0.4-μm pores. Virus expressing either EGFP or p25-EGFP was used to infect these neurons for 8-12 hours, followed by extensive washing (Figure 7A). The insert was then transferred to a culture well where adult NPC lines were seeded. These cells were co-cultured in the separated compartment mimicking the *in vivo* condition where neural progenitors coped with an environment of undergoing neuronal death. The co-culture was maintained for 1 or 3 days to examine the effect of p25-overexpressing neurons on the proliferation and survival of adult NPCs respectively. BrdU was added to the culture for 2 hours before the fixation after 1 day of culture.

No difference in the number of BrdU-positive cells was observed between WT and p25 TG mice after co-culture of isolated neural progenitors and neural stem cells (Figure 7C). The number of Ki67-negative cells was decreased in p25 TG mice (*p* < 0.01, Figure 7D), suggesting that more NPCs were in the cell cycle, and therefore, p25-overexpressing neurons likely promoted or maintained more cells in the cell cycle. After 3 days of culture, we observed that the protein level of cleaved caspase-3 was potentiated by p25 expressing neurons (Figure 7E). Moreover, a significant increase of activated caspase-3 positive cells was detected in NPC cultures (Figure 7F), consistent with the *in vivo* findings. These experiments suggested that p25 affected the proliferation and survival of NSCs in a non-cell-autonomous fashion by potentially inducing neuronal death, mimicking the effect on endogenous NPCs *in vivo*.

Wnt signaling is crucial for normal adult neurogenesis. To determine if Cdk5 regulates Wnt activity, we used the TCF reporter assay with 293T cells (supplementary Figure S3). CDK5 overexpression alone did not alter Wnt activation since 293T cells do not express p35 or p39, the regulatory subunits of CDK5. Strikingly, upregulation of CDK5 activity by co-expression of p25 or p35 suppressed TCF luciferase activity, suggesting that CDK5 activation negatively regulates the Wnt pathway.

In summary, we provided additional evidence that similarly to other AD mouse models, the CK-p25 Tg mice showed defects in adult neurogenesis and Cdk5 activation impeded the Wnt signaling pathway.

## DISCUSSION

Although the accumulation of p25 has been linked to neurodegenerative diseases such as AD [19], the effects of p25 in neurogenesis and differentiation are still poorly understood. Here, we established a transgenic mouse strain that overexpresses p25 in the postnatal forebrain and investigated the proliferation, differentiation, and survival of adult NPCs in different brain regions. Our results revealed an interesting role for p25-mediated non-cell autonomous effect in adult neurogenesis. p25 overactivation transiently stimulates NPC proliferation in both neurogenic and non-neurogenic regions, yet the upregulated NPCs are short-lived in a degenerative environment. CDK5 activation impaired the Wnt signaling pathway. This result leads to a plausible hypothesis that targeting the Wnt pathway may ameliorate aging-related decline of neural functions, especially adult neurogenesis. Therefore, intervention strategies that enhance the survival of these cells may be beneficial for neural injuries or degenerative disease.

Distinct from phenotypes observed in mice with CDK5 gain- or loss-function in adult NPCs, our study showed that p25-induction in neurons stimulated cell proliferation, especially in the non-neurogenic niche (e.g., the cortex and the hippocampal CA regions). CDK5 has a critical role in the embryonic neurodevelopment [3]. It is required for neuronal migration and correct patterning of the cortical layers. During embryonic brain development, CDK5 phosphorylates p27^Kip1^ at Thr187 and modulates embryonic NSC differentiation [29]. Consistently, knockout of CDK5 in adult nestin-expressing NPCs reduced the number of immature neurons [30]. Interestingly, either gain- or loss-of-function of CDK5 did not affect proliferation of the adult hippocampal NPCs, but rather impaired the dendritic development and synapse formation of newborn neurons in the SGZ [31]. In our mouse model, BrdU-labeled neurons were not positive for the glial cell marker GFAP, which suggests that short-term BrdU-labeled transient amplifiers instead of type B astroglial-like NSCs [32]. Even though GFAP was drastically upregulated in these mice, our data suggest that the proliferating NPCs were unlikely to be caused by reactive gliosis. Mitotic marker, ph3, confirmed that BrdU-positive cells in the brains of p25 TG mice were dividing NPCs *in vivo*. Collectively, these results suggest that overexpression of p25 in neurons causes hyperactivation of NPC proliferation in the mouse brain.

Our data demonstrated that the survival of BrdU-labeled cells is severely impaired. Widespread BrdU-labeled cell death was observed in both WT and p25 TG mice, consistent with previous observations of persistent cell death in newborn neurons, which were both age and strain specific [33]. However, p25 TG mice exhibited significantly fewer surviving BrdU-labeled cells than WT mice. Thus, p25-induced degeneration decreases the survival of proliferating progenitors despite efficiently and transiently promoting cell proliferation. BrdU-labeled cells in p25 TG mice expressed cleaved caspase-3, which is a biomarker of apoptosis, suggesting that p25-expressing neurons trigger cell apoptosis in these proliferating NPCs. These findings are consistent with previous studies that showed a critical role of p25/CDK5 in regulating neuronal apoptosis [34, 35]. Moreover, in agreement with previous study [36], most BrdU-labeled cells differentiated into both neurons and astrocytes. However, similar to the defects of newborn neurons with abnormal CDK5 activity, newborn neurons in p25 TG mice showed aberrant dendritic morphology. In addition, the lineage specification of adult NPCs was changed in p25 TG mice with more commitment to glial cells.

In the *in vitro* neurosphere formation experiment, significantly more neurospheres were observed in cells derived from the DG and cortex of p25 TG mice than control mice, but there was no significant difference in the SVZ and non-DG regions. The *in vitro* neurosphere assay amplifies slow-dividing neural stem cells and transiently amplifies NPCs. Increased numbers of neurospheres formed by cells in the DG and cortex of p25 TG mice indicate a higher number of cells that respond to mitogens in these regions. The DG harbors transient amplifiers or NPCs rather than neural stem cells [37]. Therefore, the observation of an increased number of neurospheres might be due to an increase in transient amplifiers, which would be consistent with the BrdU findings *in vivo*. Additionally, these neurospheres exhibited the ability to differentiate into astrocytes and neurons *in vitro*. Collectively, findings from the neurosphere assay support the hypothesis of hyperproliferation of neural cells in p25 TG mice, especially in non-neurogenic regions such as the cortex.

The co-culture of p25-expressing neurons with adult neural stem cells mimics the *in vivo* condition of neural progenitors surrounded by degenerative neurons. Although no significant difference in BrdU labeling was found, the percentage of Ki67-negative cells was greatly reduced in adult neural stem cells co-cultured with p25-expressing neurons. Hence, neurons overexpressing p25 consistently promoted or maintained more cells in the cell cycle. An increase in cells expressing cleaved caspase-3 was also observed. These findings suggest that p25 expressing in neurons impacts the proliferation and survival of NPCs in a non-cell autonomous fashion by potentially inducing neuronal death, mimicking the effect on endogenous NPCs *in vivo*.

In summary, we demonstrated that, in the p25 animal model, the increment of NPC proliferation was followed by a failure of these cells to survive. This is consistent with other model that response of endogenous neural stem cells to CNS insult often involves an augmented proliferation. Therefore, intervening strategies that can potentially improve the viability of endogenous NPCs may be beneficial for neural injuries or degenerative disease.

## MATERIALS AND METHODS

### Animals

p25 TG mice were established by Dr. L. H. Tsai [28]. CamK2a-tta mice (*B6;CBA-Tg(Camk2a-tTA)1Mmay/J*) from the Jackson Laboratory (Bar Harbor, ME) were used to produce the heterozygous offspring. All mice were housed in a specific pathogen-free (SPF) grade facility with food and water provided *ad libitum*. A normal circadian rhythm was adopted (12-hour light/dark cycle). CK/p25 animals were raised on doxycycline-containing food (1 mg/g in food; Bio-Serv, Frenchtown, NJ) until 6 weeks of age. To induce p25 production, mice were taken off the doxycycline diet for 6 weeks. Littermates that did not carry any transgene were used as controls and exposed to doxycycline in the same way as experimental animals. All animal experimental procedures were approved by IACUC committee of MIT and followed the national and state regulation of animal experiments.

### Immunostaining

We used the following antibody labels to mark proteins or cell events: BrdU to label proliferating NPCs, GFAP to label astrocytes, γ-H2AX to label DNA damage, ph3 to label cells at M phase, Ki67 to label cycling cells; DCX to label neurons, RIP to label oligodendrocytes, and cleaved caspase-3 to label apoptosis.

Immunolabeling was performed as described [38]. In brief, sacrificed animals were perfused with 4% paraformaldehyde (PFA). To unmask the antigen, 20-μm-thick brain sections were incubated with 4 N HCl for 20 min at 25°C. After washing three times with phosphate-buffered saline (PBS), brain sections were incubated with blocking solution containing 0.2% Triton X-100 and 5% fetal bovine serum dissolved in PBS for 1 h at room temperature. After blocking, slides were incubated with mouse anti-BrdU monoclonal antibody (1:500 dilution) alone; anti-BrdU monoclonal antibody in combination with anti-GFAP monoclonal antibody (1:500), anti-p25 monoclonal antibody (1:100 dilution), or anti-γ-H2AX antibody (1:500 dilution); and anti-ph3 antibody (1:500) in combination with anti-GFAP antibody (1:500) or anti-GFP antibody (1:500). After washing with three times of PBS, samples were probed with secondary antibody. Images were acquired with a confocal laser scanning microscope (Zeiss LSM 510) Images were further analyzed with Adobe Photoshop software (version 8.0, Adobe) and ImageJ software (v1.37, National Institutes of Health, Bethesda, MD, USA). The immune-positive cells in different brain regions were counted according to the stereology. Basically, six slices per animal were used, and 3-7 animals were used for each genotype group. In Figure 4A, we further divided the SVZ into sub-regions, including the lateral or dorsal walls. Both walls were further divided into rostral and caudal portions using the boundary of emerging point of hippocampal proper as a landmark.

### BrdU injection

BrdU injection was conducted as described [39]. Briefly, after 6 weeks of induction, animals received intraperitoneal injections of BrdU at a concentration of 50 μg/g body weight. For pulse labeling, BrdU was administered three times per day (at 2-hour intervals) within a day. For prolonged labeling, BrdU was injected twice per day (4 hours apart) for 3 consecutive days. Animals then were sacrificed on either Day 0 or 7 according to the experimental design for immunostaining.

### Primary culture of cortical neurons

Primary culture of mouse cortical neurons was carried out as described [38]. Briefly, cortical tissues were dissected from the embryonic mouse brain (embryonic day 15). Cortical tissues were minced into 1-mm^3^ pieces and then dissociated by 0.05% trypsin-DNase solution for 15 min. After digestion, tissues received mechanical trituration and were prepared as single-cell suspensions in neural basal medium containing 2% B27 supplement, 0.2 mg/ml NaHCO_3_, 20 mM D-glucose, 2 mM glutamax, 25 U/ml penicillin, and 25 U/ml streptomycin. Cells were seeded onto a culture dish at a density of approximately 8 × 10^4^ −1.2 × 10^5^ cells/cm^2^. Cells were maintained at 37°C in a 5% CO_2_-humidified incubator.

### Primary culture of neuronal stem cells

#### Neurosphere assay

Specific regions, including neocortex, the SVZ/VZ lining of the lateral ventricles, and the DG and non-DG regions, were dissected from the brains of WT and p25 TG mice [40]. Cells from these regions were dissociated and grown free-floating in NPC culture medium. After 7 days, the number of neurospheres were counted, standardized, and compared between different regions.

### Transwell co-culture system

The 0.4-μm pore Transwell polycarbonate membrane inserts were pre-coated with poly-D-lysine and laminin. Primary neurons isolated from WT or p25 TG mice were seeded onto the membrane on the upper inserts at a density of 10,000 cells/well and maintained in fresh Neurobasal culture medium. At 24 h after seeding, 1 μl of herpes simplex virus (HSV) expressing enhanced green fluorescent protein (EGFP) or p25-EGFP was added to the culture medium to infect neurons. After 6-8 hours of infection, the virus-contained medium was removed, and the cell culture was washed extensively three times with PBS. To determine the efficacy of the infection, the expression of EGFP in infected cells was examined under a fluorescent confocal microscope (LSM 5, Zeiss). After confirmation, the inserts were transferred to a 6- or 24-well culture dish on which adult neural stem cells had already been seeded at medium density. The co-cultures were maintained in the neural stem cell culture condition for 1-3 days. Adult neural stem cells co-cultured with primary neurons in 6-well plates were lysed, and proteins were collected for western blotting analysis. Adult neural stem cells seeded on coverslips and co-cultured with primary neurons in 24-well plates were fixed in 4% PFA and probed with specific antibodies for immunocytochemical analysis. For BrdU staining, BrdU was added into the culture medium at a final concentration of 10 μM 2 h before 4% PFA fixation. The immunostaining and BrdU staining procedures were conducted as described above.

### Western blotting

Total protein was extracted from neural stem cells using RIPA lysis buffer containing 50 mM Tris-HCl (pH 7.4), 150 mM NaCl, 1% NP-40, and 0.1% SDS. The protein concentration in different extracts was measured using the Bradford assay. Equal amounts of protein samples were separated by sodium dodecyl sulfate-polyacrylamide gel electrophoresis. Then, proteins were transferred to a PVDF membrane (Millipore). The PVDF membrane was then blocked with 5% w/v non-fat dry milk dissolved in Tris buffered saline plus 0.1% Tween-20 (TBS-T), followed by primary antibody incubation at 4°C overnight. The primary antibodies used in this study were anti-cleaved caspase-3 (1:500 dilution) and anti-α-tubulin (1:1000 dilution). The housekeeping protein α-tubulin was used as an internal control. After washing three times with TBS-T, membranes were incubated with HRP-labeled secondary antibodies (1:2000 dilution) for 2 h at room temperature. Immunobands were detected using an enhanced chemiluminescence (ECL) kit according to the manufacturer's instructions (Millipore).

### Luciferase assays

5×10^5^ 293T cells were seeded into 24-well plates and 0.5 μg of vector, CDK5 plasmid, CDK5 plus p25 or p35 plasmid was transfected with 0.1 μg of Super8XTOPFLASH and 20 ng of pRL-TK using Lipofectamine 2000 (Invitrogen). At 24 h after transfection, transfected cells were stimulated with Wnt3a-conditioned medium (Wnt3a CM) for 14 h, and TCF reporter activity was measured using the Dual-Luciferase Assay System (Promega). All firefly luciferase activities were normalized with Renilla luciferase activity.

### Statistical analysis

Data were analyzed in Statistical Package for the Social Sciences software (version 11.0, SPSS Inc., Chicago, IL, USA). All bar graphs were plotted as the mean ± standard error of the mean (SEM). Measurement data were tested by Student's *t*-test (for two groups). *p* < 0.05 was considered statistically significant.

## SUPPLEMENTARY MATERIAL FIGURES


