# Supplementary material for: Transient enhancement of proliferation of neural progenitors and impairment of their long-term survival in p25 transgenic mice
Source: Oncotarget. 2016 Jun 6;7(26):39148–61. doi: 10.18632/oncotarget.9834 (PMC5129921; doi:10.18632/oncotarget.9834)
Supplement: Supplementary file 1 [file oncotarget-07-39148-s001.pdf]

Transient enhancement of proliferation of neural progenitors and impairment of their long-term survival in p25 transgenic mice

Supplementary Material

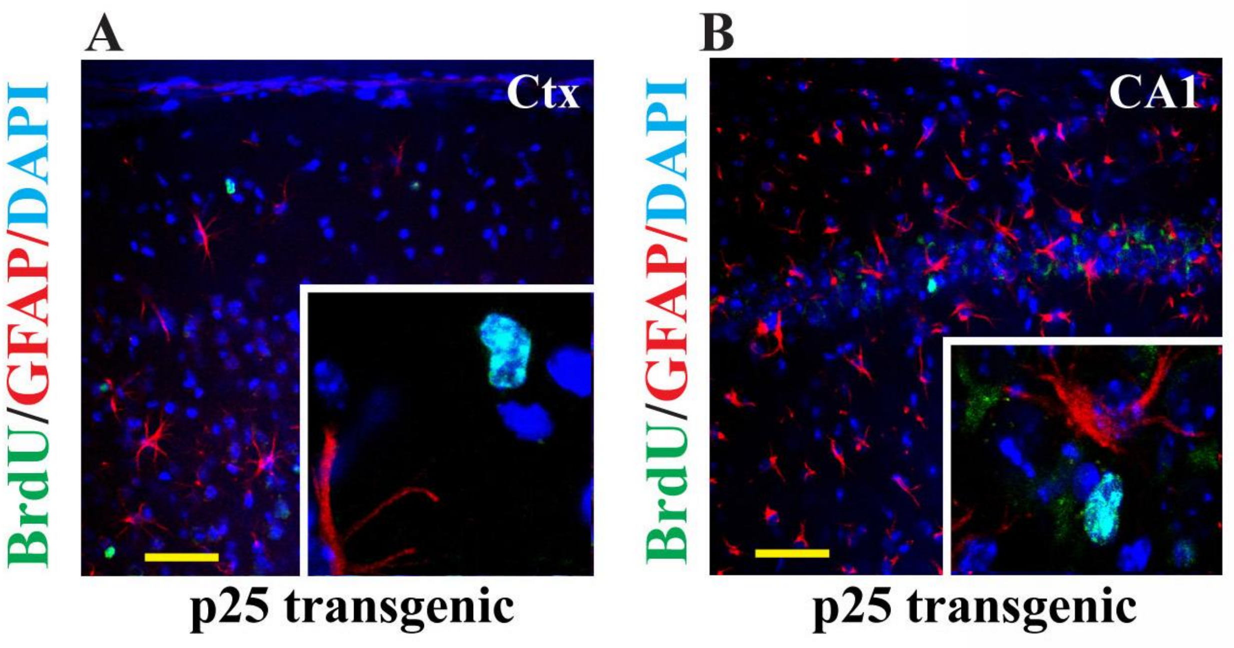

Supplementary Figure S1. BrdU positive cells in the cortex (A) and the CA1 region (B) of CK-p25 mice are not GFAP-positive astroglial cells. Scale bar= 50 µm.

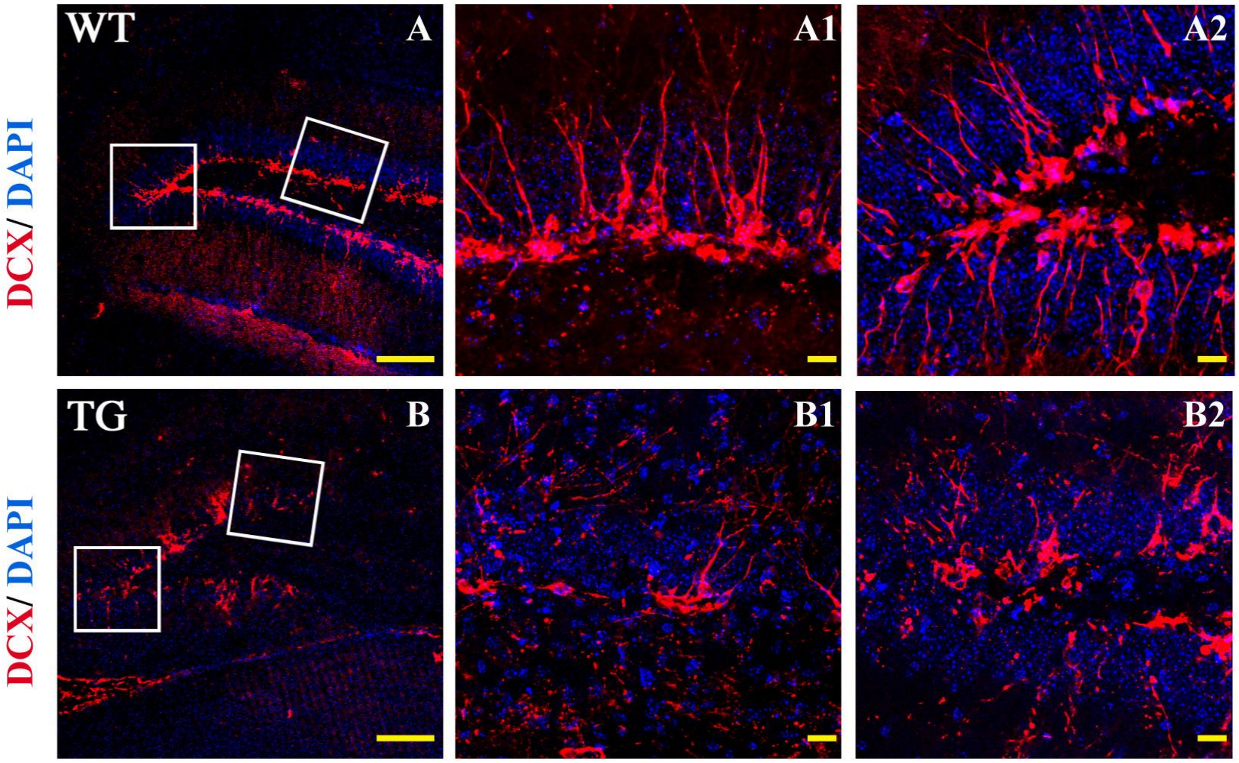

Supplementary Figure S2. Long-term survival of BrdU-labeled neuronal progenitor cells *in vivo*. After 6 weeks of induction, the number of DCX-labeled (red) cells was reduced in the brains of control (A, A1, A2) and p25 TG (B, B1, B2) mice. Nuclei were counterstained with DAPI (blue). Scale bar= 50 μm in A and B; Scale bar= 10 μm in A1-A2 and B1-B2.

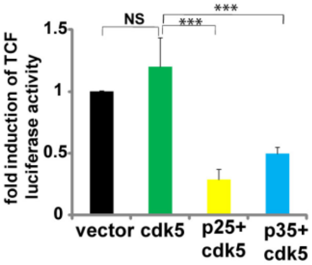

Supplementary Figure S3. CDK5 activation impaired Wnt activation. ***, $p < 0.005$, n=3. NS, not significant.
